# Reagentless Vis-NIR Spectroscopy Point-of-Care for Feline Total White Blood Cell Counts

**DOI:** 10.3390/bios14010053

**Published:** 2024-01-19

**Authors:** Teresa Guerra Barroso, Carla Queirós, Filipe Monteiro-Silva, Filipe Santos, António Hugo Gregório, Rui Costa Martins

**Affiliations:** 1 1H-TOXRUN—One Health Toxicology Research Unit, University Institute of Health Sciences (IUCS), CESPU, CRL, 4585-116 Gandra, Portugal; teresa.barroso@iucs.cespu.pt; 2 LAQV-REQUIMTE, Faculty of Sciences, University of Porto, R. Campo Alegre, 4169-007 Porto, Portugal; carla.queiros@fc.up.pt; 3 INESC TEC—Institute for Systems and Computer Engineering, Technology and Science—Campus da FEUP, Rua Dr. Roberto Frias, 4200-465 Porto, Portugal; filipe.m.silva@inesctec.pt (F.M.-S.); filipe.n.santos@inesctec.pt (F.S.); 4 Anicura CHV—Veterinary Hospital Center, R. Manuel Pinto de Azevedo 118, 4100-320 Porto, Portugal; antonio.gregorio@iucs.cespu.pt

**Keywords:** point-of-care, spectroscopy, white blood cells, artificial intelligence

## Abstract

Spectral point-of-care technology is reagentless with minimal sampling (<10 μL) and can be performed in real-time. White blood cells are non-dominant in blood and in spectral information, suffering significant interferences from dominant constituents such as red blood cells, hemoglobin and billirubin. White blood cells of a bigger size can account for 0.5% to 22.5% of blood spectra information. Knowledge expansion was performed using data augmentation through the hybridization of 94 real-world blood samples into 300 synthetic data samples. Synthetic data samples are representative of real-world data, expanding the detailed spectral information through sample hybridization, allowing us to unscramble the spectral white blood cell information from spectra, with correlations of 0.7975 to 0.8397 and a mean absolute error of 32.25% to 34.13%; furthermore, we achieved a diagnostic efficiency between 83% and 100% inside the reference interval (5.5 to 19.5 × $10^{9}$ cell/L), and 85.11% for cases with extreme high white blood cell counts. At the covariance mode level, white blood cells are quantified using orthogonal information on red blood cells, maximizing sensitivity and specificity towards white blood cells, and avoiding the use of non-specific natural correlations present in the dataset; thus, the specifity of white blood cells spectral information is increased. The presented research is a step towards high-specificity, reagentless, miniaturized spectral point-of-care hematology technology for Veterinary Medicine.

## 1. Introduction

**Figure 1:**
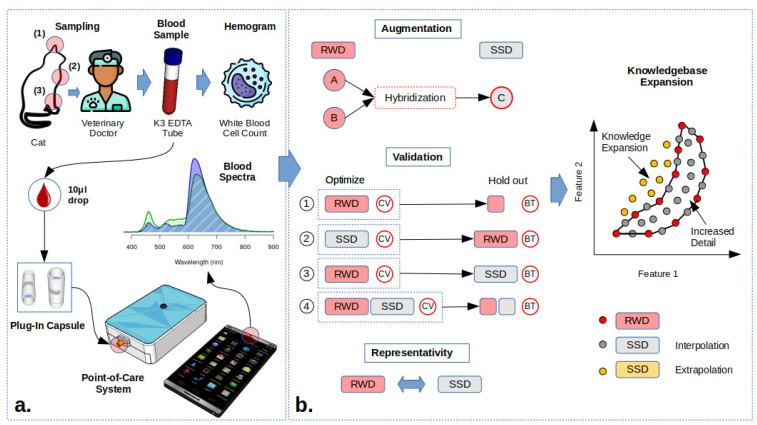
Total white blood cell counts: (**a**) sampling and spectroscopy procedures—venipuncture performed at the jugular (2) or cephalic (3) veins for hemogram analysis and single drop for POC spectral recording; a single drop can also be collected at the auricular vein (1). (**b**) Data augmentation through hibridization of real-world data (RWD) into synthetic spectroscopy data (SSD), validation procedures using RWD and SSD as optimization and validation datasets to test SSD representativity of RWD and knowledgbase expansion.

Blood spectra are governed by multi-scale interference and matrix effects, super-imposing physical (scattering) and chemical (absorbance) information. Unscrambling the information contained in spectral data, due to multi-scale interference, is the major challenge for point-of-care (POC) technologies [1,2] (Figure 1a). Multi-scale interference results from overlapping spectral bands of blood constituents, leading to interference at varying intensities and deviations from the Beer–Lambert law (BLL) [3]. Matrix effects are inherent to sample properties, such as pH or scattering effects, affecting the proportions of Mie and Rayleigh scattering and absorbance bands [4,5]. Clinical and analytical chemistry address spectral interference through sample composition simplification (e.g., separation using filtration, centrifugation, chromatography) or reaction specificity on biochips (e.g., immunological reactions, molecularly imprinted polimers, aptamers), not taking advantage of the rich information present in spectra [6,7,8,9,10].

Unscrambling the multi-scaled interference and matrix effects, and unlocking constituent-specific information in each spectrum, lies at the heart of reagentless POC technology [1,11,12]. The use of artificial intelligence in biosensor development and operation is currently a reality [13,14]. Significant advances in searching for consistent covariance modes capable of isolating both inference and constituents information has allowed for the provision of information equivalence between spectra and hemograms, leading to the precise quantification and diagnosis of clinical conditions [1,11,12,13,14,15,16] and demonstrating the possibility of quantifying white blood cells (WBCs) in dog blood [2]. Herein, we explore the quantification of WBCs in cat blood through the isolation of specific WBC information in blood spectra, taking advantage of the feline physiology for spectral quantification, such as the use of scattering effects of WBCs vs. red blood cells (RBCs) and avoiding the use of inferential WBC quantification, such as the natural relationship between RBC and WBC levels.

### 1.1. White Blood Cells and Blood Spectroscopy

The measurement of WBCs is crucial in hematology due to its wide-ranging diagnostic significance, encompassing conditions like infections and leukemia. The main reasons for leucocytosis (increased WBC count) are inflammation, effects of stress, corticosteroids, physical activity, and presence of epinephrine [17]. High WBC counts are mostly associated with neutrophilia and inflammation, whereas neutropenia is mainly caused by increased peripheral tissue demand during intense inflammation and/or decreased bone marrow production [18]. Due to a substantial reservoir of neutrophils in the bone marrow, dogs and cats rarely experience leukopenia (low WBC count) [17].

Blood spectra information is dominated by the strong absorbance of hemoglobin (Hgb) [19] and bilirubin (Bil) [20]. RBCs dominate spectral fingerprinting and the presence of interferents must be taken into account, e.g., Bil interfering with Hgb-related features [1,19].

WBCs are traditionally measured by electrical impedance, laser light scattering, radiofrequency conductivity and flow cytometry [21]. Automated hematology analyzers rely on cell size, which impacts both impedance and scattering angle measurements. Therefore, development stage variations in blood cell sizes, and platelet (PLT) clumping [22], are the main reasons for inaccuracies in modern automated hematology systems [23,24,25]. To check the automated results, microscopy blood smear counting is applied as a gold standard quality control measure in clinical practice [25], which also allows for the analysis of nuclei appearance, cell size, cytoplasmic granules and toxic cytoplasm [26,27].

Spectroscopy is not limited by sample volume or reagents, making it well-suited for portable POC technology. Figure 1a showcases the prototype system, which integrates Internet of Things (IoT) electronics and software, controllable through a smartphone without the need for a dedicated app. A single drop of blood (10 μ L) is placed in a reusable capsule designed for insertion into the transmittance probe tip [1] (Figure S1). These capsules are constructed with opposing mirrors to optimize internal reflections and light is collected via a central pinhole fiber optic connected to the spectrometer that operates within the 300–800 nm wavelength range [1].

Ultraviolet–visible (UV-Vis) spectroscopy distinguishes the different organelles of leucocytes, including cytosol, nuclei, mitochondria and membranes [28]. Changes in absorbance (200–400 nm), fluorescence/phosphofluorescence and infrared patterns of WBCs reflect significant alterations in organelle composition, allowing for the diagnosis of leukemia [29,30,31,32,33] and its progression [34,35,36,37]. Infrared microscope spectroscopy of WBC components was used to diagnose hidden infections through support vector machines classification [38]. These studies show the possible existence of unique spectral features associated with WBCs, which allows us to overcome the problem of low quantities of WBCs in comparison to RBCs—a ratio of approximately 1:1000.

Feline Hgb has a lower affinity to oxygen (and is more readily uptaked by tissues) [26], resulting in a lower packed cell volume (PCV) and Hgb concentration than in canines [39]. Feline PLT are larger and exhibit greater size variation (15 to 18 μ m) in comparison to other domestic mammals [26]. In specific situations, there can be a strong correlation between RBCs and WBCs, and special care must be taken because optimal chemometrics/AI models can be dependent on intrinsic data correlations and a lack of causal connections to constituent absorbance [40].

Reagentless spectral POC demonstrated its effectiveness in quantifying hemogram parameters in dog and cat blood [1,2]: (i) Dog blood—RBCs (6.39%), Hgb (7.14%), hematocrit (HTC) (4.43%); (ii) cat blood—RBCs (5.67%), Hgb (4.08%), and HTC (1.69%). WBCs in dogs were quantified with a correlation of 0.8478, standard error of 6.92 × $10^{9}$ cells/L and MAPE of 25.37%, allowing us to diagnose WBCs in the reference interval (5.6–17.8 × $10^{9}$ cells/L) [2].

In pure constituents, the signal varies uni-dimensionally along a single eigenvector, as it has only one source of variance—a covariance mode (CovM). CovMs isolate the interference between constituents by searching for a group of samples that maximizes the variance of the constituent being quantified [1,12]. In special cases, CovMs can be orthogonal, isolating specific and unique information [40]. Due to the significant biological variability, the covariance of large representative datasets becomes unstable and exhibits high dimensionality. It becomes necessary to unscramble various types of interference to accurately relate the quantitative information of a specific constituent within the context of its interferents through CovMs [12].

Each sample within a CovM exhibits a consistent covariance between spectral information ( X ) and constituents ( Y ). This implies the two blocks of information are similar, despite presenting with different bases (wavelengths vs. concentration). Consequently, the two information blocks demonstrate latent structural similarity ( t∼u ), where t and u are derived independently via the singular value decomposition of X and Y : $X=TP^{t}$ and $Y=UQ^{t}$ . Here, P and Q are the orthogonal bases of T and U , respectively. Ideally, within each CovM, the interference information is equivalent to the concentration ( t ∼ u ), represented by a single eigenvector or one latent variable (1 LV). This offers a causal interpretation of spectral interference by associating the absorbance bands of constituents [3] with the BLL law relationship [1,12].

### 1.2. Data Augmentation

Data augmentation can be used to enhance the diversity of the knowledgebase and improve the accuracy of model predictions [2,41,42]. Blood composition representativity is limited in real world data (RWD) due to the large biological variance. This is partially solved by self-learning models, which can assess if the measured spectra are predictable, suggesting an update to the knowledgebase [12]. Synthetic spectroscopy data (SSD) are generated by the random hybridization of the hemogram and spectral information from two samples (Figure 1b), creating new spectra and a corresponding hemogram with a 1:1 information mixture, which is comparable to the mixture of the two blood samples, resulting in the average composition of the two RWD samples and assuming no reaction between blood samples (e.g., antigen–antibody of blood type and Rhesus factor) [2]. SSD improves the knowledgebase by (i) increasing the level of local detail by the interpolation of the existing RWD samples and (ii) expanding the knowledgebase through extrapolation, once the boundary coordinates of the samples in the feature space are convex (Figure 1b).

The objective of this research is to demonstrate the feasibility of spectral WBC quantification in cat blood by (i) demonstrating the capacity of SSD to both represent and increase the level of detail of the RWD knowledgebase; (ii) benchmarking the global covariance through partial least squares (PLS) versus local covariance through Local-PLS (LocPLS) and Self-learning Artificial Intelligence (SLAI); and (iii) determining the WBC diagnostic potential of spectral POC through bias-variance analysis inside and outside the reference interval (RI).

## 2. Methods

### 2.1. Hemogram Analysis

Blood samples from cats, which are already employed in clinical diagnostic procedures, were obtained from the jugular vein through standardized venipuncture procedures conducted by qualified personnel at the Anicura CHV—Veterinary Hospital Center. The remaining blood from EDTA tubes, collected previously and still in a fresh state, was subsequently utilized for these tests. Hemogram parameters were determined using the Mindray BC-5800-vet auto-hematology analyzer (Mindray, Shenzhen, China) [43] (Figure 1a).

### 2.2. Spectroscopy

Figure 1a illustrates the Visible Short-Wave Near-Infrared (Vis-SWNIR) POC and IoT prototype platform [44]. It uses a Hamamatsu C12666MA spectrometer that manages light sources (LED or laser diodes). This version uses a power LED (4500 K) as a light source (Figure 1a). Optical configuration used a transmittance probe with six illuminations and a central collection fiber. Blood is analyzed inside a plug-in capsule with an opposing mirror configuration (5 mm path) (see Figure S1). The average of three blood spectra is recorded and spectra are corrected for scattering effects before further processing [45]. A total of 94 blood samples from different cats were used in this study for hemogram and spectral records.

The spectral POC operation is exemplified in Figure S1, with the following steps: (1) withdrawal of 10 μ L of blood from the ear lobe; (2) injection of blood sample into the capsule system; (3) plug-in of the capsule into the POC probe; and (4) use of IoT software to control POC measurement and record spectra for WBC calculation.

### 2.3. Spectral Data Augmentation

A synthetic dataset is created by the hybridization of two random RWD samples. Hybrid samples do not exist in the RWD, and may or not reflect existing blood samples in feline species (Figure 1a). However, the combination of information promotes knowledgebase expansion by spawning spectral gradients proportional to blood hemograms. A total of 300 SSD samples were obtained by non-repeated random mixing of pairwise RWD spectral samples ( X ) and hemogram compostion ( Y ), where $X_{SSD}=\frac{1}{2}\left(X_{i}+X_{j}\right)$ and $Y_{SSD}=\frac{1}{2}\left(Y_{i}+Y_{j}\right)$ , with *i* and *j* random samples. Hybridization creates a synthetic sample with average spectral ( $X_{SSD}$ ) and blood composition ( $Y_{SSD}$ ) characteristics, where each pixel in the spectra is the average pixel intensity of $X_{i}$ and $X_{j}$ real-world spectra at a given wavelength, and the $Y_{SSD}$ WBC value is the average value of two real-world hemogram WBC values, $Y_{i}$ and $Y_{j}$ .

Figure 1b describes how SSD was tested for RWD representativeness (null hypothesis) by spliting model optimization by cross-validation (CV) and blind testing (BT): (1) RWD benchmark leave-one-out optimization and leave-one-out holdout blind testing in PLS models (RWD); (2) SSD optimized—leave-one-out CV optimization and blind testing with RWD (SSD>RWD); (3) optimized leave-one-out CV optimization and BT testing with SSD (RWD>SSD); and (4) joint RWD and SSD and random splitting into optimization using CV and BT datasets (90:10) (Figure 1b, RWD + SSD). Rotation to RWD and SSD datasets in schemes 1 (RWD) and 4 (RWD+SSD) was performed during optimization, so that all samples could be evaluated in BT. The increase in the information in a two-dimensional feature space via data augmentation is graphically represented in Figure 1b. RWD restricts the knowledgebase to a polygon area within the feature space (Figure 1b). Inside the polygon, the SSD is generated using the RWD samples at the edges, resulting in an increase in the representativeness of the local covariance.

Extrapolated samples in yellow in Figure 1b are obtained through the hybridization of the convex boundary samples of the RWD polygon (Figure 1). Extrapolated SSD retains the information on spectral gradients and interferences to be proportional to nutrient composition, extrapolating unknown characteristics.

### 2.4. Benchmarking

CovM search methodology was benchmarked against chemometrics methods:

- Partial least squares regression (PLS): PLS maximizes the covariance between the spectra matrix X and the blood hemogram matrix Y , by determining the eigenvectors of $X^{t}Y$ [46,47]. The method forces the latent structures of spectra and hemogram ( t and u ) to be equal (NIPALS or SVD algorithm), for the correspondent basis $P^{t}$ and $Q^{t}$ [46], where $X=TP^{t}$ and $X=UQ^{t}$ . It further deflates sequential orthogonal eigenvectors of the remaining information in $X^{t}Y$ . The number of latent variables (LV) is optimized by the cross-validation of the minimal predicted sum of squares. PLS uses an oblique projection to determine the $b_{pls}$ coefficients in $Y=Xb_{pls}$ , which, albeit it linear combination of LVs, can model spectral information non-linearities [47,48].
- Local PLS (LocPLS): breaks down the complexity of the feature space by KNN classification based on the similarity of spectral features given by the Euclidean distance between sample coordinates. An ensemble of PLS models is generated for each cluster. The dimensions of the feature space and number of the cluster was optimized by cross-validation/hold-out samples [49].
- Self-learning Artificial Intelligence (SLAI): uses a two-step approach that (a) features space optimization for providing equivalence between the spectral information ( T ) and blood hemogram ( U ) latent spaces; and (b) performs a local covariance mode (CovM) search for unscrambling groups of samples that have the same type of interferences, providing a direct quantification between spectra and nutrients with a single dimension (or eigenvector) exhibiting stable co-variance ( $X^{t}Y$ ) [1].

A two-step approach was used to build and validate the models: (i) model optimization through cross-validation using a training set; and (ii) prediction and corresponding metrics using blind testing samples. Local models (LocPLS and SLAI) use the leave-one-out CV with one hold-out sample in the model optimization step. The performance was evaluated according to the standard deviation (SE), mean absolute percentage error (MAPE), and Pearson correlation (R), as the linearity between WBC and predicted values. CRAN-R software was used for all computations (PLSR package; LocPLS and SLAI using the author’s code) [50].

### 2.5. Total Error and Bias Analysis

The American Society for Veterinary Clinical Pathology (ASVCP) establishes the total allowable error (TAE) of the clinical decision target values (TVa), which, in the case of hemograms, are the boundary values of the Reference Interval (RI, 5.5 to 19.5 × $10^{9}$ cell/L) [25], with a maximum TV = 23.7 × $10^{9}$ cell/L. The distribution of the total error (TE) results in ASVCP classes of optimal 7.15% (opt), desired 14.29% (des) and acceptable 21.45% (acp), which were used as bias performance indicators [51]. The percentage of correct diagnoses with increasing WBC count was used to assess the diagnostic capacity of the spectral POC.

## 3. Results and Discussion

### 3.1. WBC Blood Spectroscopy

**Figure 2:**
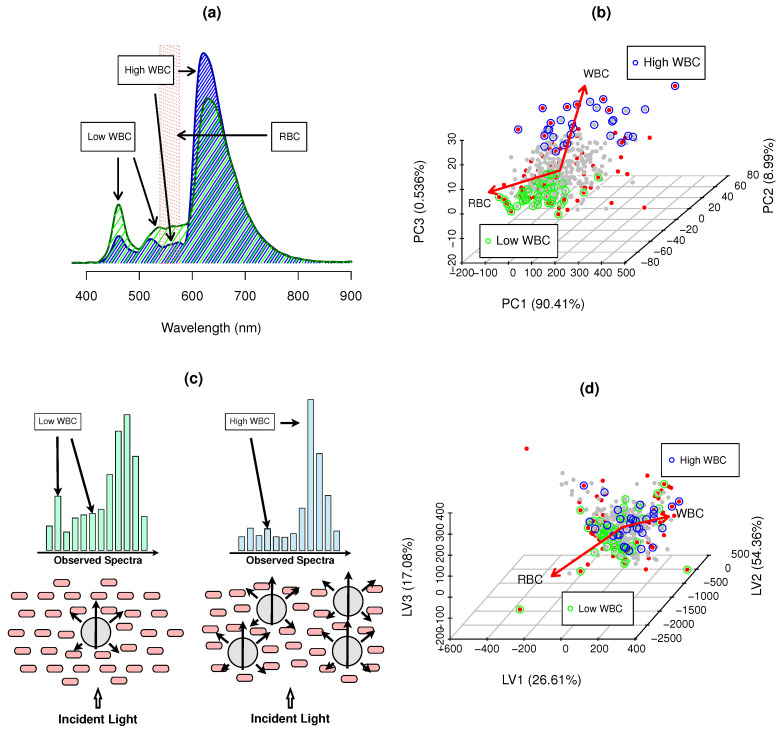
WBC spectral information: (**a**) cat blood spectra (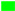) low WBCs, (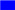) high WBC examples, and (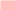) RBC Hgb major inteference bands (539–576 nm); (**b**) PCA scores of hemogram counts; and (**c**) Cell packing, scattering and WBC absorbance effects and impact on observed spectra; (**d**) PLS scores of blood spectra—translating maximum covariance to WBCs, where • SSD blood samples, • RWD blood samples, • low WBCs and • high WBCs and → hemogram PCA loadings and main gradient direction in PLS scores space.

Figure 2a presents two typical blood spectra with low and high WBC levels. WBC spectral information is spread along the wavelengths 400–800 nm. WBC increase leads to an increase in absorbance in the region of interest from 400 to 600 nm (ROI 1) and a decrease in absorbance from 600 to 800 nm (ROI 2). WBCs and RBCs/Hgb are interferents because several species of Hgb absorb in ROI 1 and 2, with higher intensity in the region of 530 to 576 nm, and with smaller contributions at 430 nm and 630 nm, with the following hemoglobin absorption bands: (i) HbA—433 nm and 558 nm; (ii) Oxy HbA—539 nm; (iii) Carboxy HbA—550; (iv) Ferrous HbA—541 nm and 576 nm; (v) Deoxy HbA—555 nm; (vi) Ferryl HbA—545 nm; and (vii) Ferric HbA—630 nm. [1,19,52]. Similar results were observed in dog blood, where ROI 1 and ROI 2 showed similar changes in absorbance for high and low levels of WBCs, with a more significant interference from RBCs/Hgb in dogs [2].

The scattering ratio of RBCs and WBCs can be defined as S=2πR/λ , where *R* is the particle radius and λ the wavelength, Cat RBCs and WBCs have a diameter approximately of 5.5 μ m and 18 μ m, and are scattering dominated by geometric effects (S >> 1), which partially explains the observed spectra (Figure 2c).

Despite of the larger number of RBCs in cats, these are smaller and contain less hemoglobin. The significant size discrepancy between WBCs and RBCs provides WBC information in blood spectra due to the increased scattering and absorption (Figure 2c). The scattering ratios of RBCs at 400 and 800 nm were 86 and 43, whereas those for WBCs were 282 and 143. WBC presence in the blood leads to a significant decrease in the observed spectral intensity, which is more relevant in ROI 1. As scattering is less intense at higher wavelengths, ROI 2 is significantly less affected by WBCs and RBCs, whereas with more WBCs, the signal increases in ROI 1 (Figure 2a,c).

The surface exposure to light of WBC is ∼213.71 μ m $m^{2}$ , whereas, in RBC is ∼23.75 μ $m^{2}$ , the cell thickness of WBCs (∼10 μ m) is significantly higher than anucleated RBCs (∼2 μ m). The smaller area, thickness and lack of nuclei leads to more space between RBCs at low WBC levels (Figure 2c). High levels of WBCs significantly increase the absorbed light, as WBCs increase the exposed surface and light path length, as well as the cell packing in smaller spaces within the blood (Figure 2c). This results in light exposure ratios of 0.5% for combinations of low WBCs (5 × $10^{9}$ cell/L) and high RBCs (9 × $10^{12}$ cell/L), and 22.5% for combinations of high WBCs (50 × $10^{9}$ cell/L) and low RBCs (2 × $10^{12}$ cell/L). The absorbance ratio also increases at high WBC levels, as the light path length through cells can increase three to five times, supporting the reason why WBC information is present in the spectra.

RGB and WBC packing limitations impose a physical restriction, resulting in the intrinsic negative correlation between RBC and WBC latent spaces (Figure 2b,d). As WBC levels increase in abnormally high levels, the available free plasma volume for RBCs decreases, which imposes a natural negative correlation, which, despite being statistically valid, has no relation to specific WBC spectral information.

Figure 2b,d present the first three scores of the hemogram PCA analysis and spectral WBC PLS. PCA calculates the orthogonal eigenvectors that maximize the variance of the hemogram, whereas PLS maximizes the covariance between the spectra ( X ) and WBCs ( Y ) by extracting the orthogonal eigenvectors of the covariance matrix ( $X^{t}Y$ ). The quantification of WBCs is only feasible if the spectral information has a similar eigenstructure to the hemogram variance space. If X is the spectra (samples × wavelength), then Y is the hemogram (samples × [RBC, Hgb, HTC, WBC, PLT]), being decomposed into: $X=TP^{t}$ and $Y=UQ^{t}$ , where T and U are the coordinates (aka scores), $P^{t}$ and $Q^{t}$ (aka loadings). Once X and Y share significant common equivalent information, their eigenstructures must also reflect such similarities. Therefore, it is expected that T and U have a similar structure and sample distribution along their space coordinates, despite being expressed by different bases $P^{t}$ and $Q^{t}$ ; this is because spectra carry hemogram information ( T≃U ).

Hemogram variance space U is presented in Figure 2b, where 99.94% of the variance is represented by three principal components, PC1 (90.41%), PC2 (8.99%) and PC3 (0.536%), where high levels of RBCs (8–10 × $10^{12}$ cell/L) and WBCs (20–50 × $10^{9}$ cell/L) dominate hemogram variance. RBCs and WBCs in the cat blood hemogram increase in opposite directions and are not orthogonal, exhibiting the natural negative correlation that is expected in the interaction of cell packing, scattering and absorbance effects, as shown by the main variance vectors (red arrows, Figure 2b).

Spectra have more information than blood hemograms, as they reflect both cellular and serum fractions. This affects the structure of T (Figure 2d). Only the WBC information should be used in the quantification provided by similarity ( T≃U ) at each CovM. T (Figure 2d) has a high degree of similarity with the hemogram space U . Three LVs represent 98.77% of the covariance with WBCs (LV1 (26.94%), LV2 (54.36%) and LV3 (17.08%)). High WBC values also showed a negative correlation with high RBCs, where the main direction vectors were non-orthogonal (Figure 2d), maintaining a similar relationship to that shown in the hemogram space ( U ) (Figure 2b). The structural similarity between T and U shows the hemogram and spectral information are similar at both global and local variance levels, allowing us to relate WBCs to spectral variance on both scales [1,2,12].

### 3.2. Significance and Representability

A significant challenge for the spectral quantification of non-dominant constituents, such as WBCs, is to establish a well-balanced representation of the knowledgebase, with a detail level allowing the isolation of consistent small-scale spectral variance. In this study, the RWD provides a low local representation of the global covariance ( $X^{t}Y$ ), as the *p*-value of PLS-RWD is 0.0001. Blood spectra covariance is highly structured, with significantly high levels of locally stable co-variances, leading to the existence of multiple interference modes [1,2]. PLS-RWD can only relate the major spectral covariance with 2LVs to WBC. The PLS model averages the existing local covariances, but is not able to extract further LVs due to the lack of local representativeness, mostly capturing the natural negative correlation of WBCs and RBCs due to cell packing and scattering.

The results show that SSD provides a stable global covariance matrix ( $X_{SSD}^{t}Y_{SSD}$ ), allowing the PLS algorithm (PLS-SSD>RWD) to extract 15 LVs with a *p*-value of 1.12 × $10^{-5}$ . The large number of LVs is consistent with a significant number of locally stable interferences. Another proof of SSD representativity of RWD is SSD prediction using the PLS-RWD>SSD model (*p*-value < 2 × $10^{-16}$ ), which leads to the conclusion that RWD and SSD represent the same information (Table 1, Figure S2).

Local models (LocPLS, SLAI) with SSD>RWD attain significant *p*-values (4.16 × $10^{-6}$ and <2 × $10^{-16}$ , Table 1). Local representativity is reflected in the lower number of LVs shown by LocPLS (5 LVs) and SLAI (1–2 LVs), resulting in a higher efficiency in isolating interference and accuracy in WBCs. Augmentation through hybridization increases local information by creating samples with mixed properties, complementing the information of the under-represented coordinates of the feature space (Figure 1b and Figure 2c,d). The statistical significance of SSD representativity shows that hybridization is an efficient method for increasing knowledgebase information. Further evidence of the increased information provided by SSD can be seen when RWD and SSD are used together. PLS, LocPLS and SLAI show significant *p*-values (<2 × $10^{-16}$ , Table 1). RWD has a significant diversity of information for SSD combinations that can improve local representativity.

### 3.3. WBC Quantification

The PLS-RWD has a low correlation (R = 0.1874) and a high MAPE (55.45%). The low correlation and number of LVs (2 LVs) shows that PLS-RWD lacks the local detailed information to enable the incorporation of WBC characteristics into more LVs. When PLS-RWD is used to predict SSD, a low correlation (R = 0.2634) and high error (MAPE = 44.60%) are obtained (Table 1).

R and MAPE improve significantly when SSD is used to predict RWD. PLS-SSD>RWD exhibits an R = 0.5775, MAPE = 44.56% and five LVs. A similar result is achieved when RWD and SSD are used in the PLS-RWD+SSD model (R = 0.5573, MAPE = 57.31%) using 13 LVs (Table 1). A higher number of LVs occurs due to the increase in the detail of the local relationship between T and U in the SSD dataset. WBC information is present in low-intensity variation in the local spectra, as WBC information is present in low-intensity variation in local groups.

The use of SSD in local models improves WBC quantification. LocPLS-SSD>RWD improves WBC quantification (R = 0.4546, MAPE = 58.89%), reducing the number of LV to five. LocPLS SSD prediction is better than RWD. RWD has abnormally high WBC values, which are under-represented in SSD. Hybridization is not very efficient for solving class imbalance at extreme values. Using SSD and RWD as the knowledgebase mitigates this problem. The LocPLS-RWD and SSD model has less extreme values of class imbalance, and, therefore, RWD prediction is significantly improved (R = 0.7112, MAPE = 46.78%), even when compared with SSD (R = 0.6782, MAPE = 32.16%), without a significant increase in LVs (seven LV).

**Figure 3:**
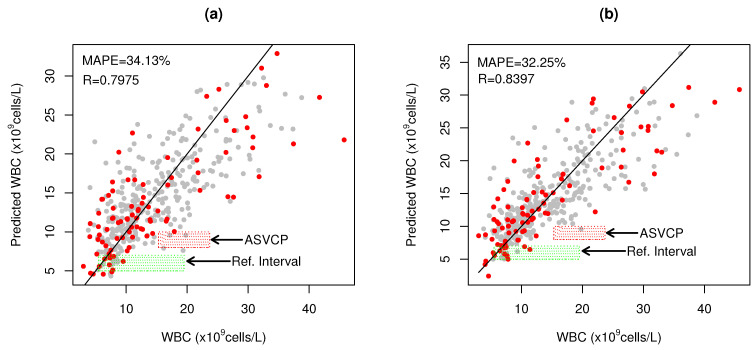
WBC prediction for (**a**) SLAI with SSD as CV optimization and RWD as HO blind test samples; (**b**) SLAI with RWD and SSD as CV and HO samples, where (•) represent the hybridized SSD samples and (•) the RWD blood samples, respectively. Green shaded rectangle (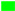) represents the WBC reference interval for cats (5.5–19.5 × $10^{9}$ cells/L) and red shaded rectangle (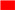) represents the ASVCP total allowable error tolerance for high WBC diagnosis.

SLAI-SSD>RWD has consistent results between SSD (R = 0.7369, MAPE = 22.35%) and RWD (R = 0.7975, MAPE = 34.13%), with only one to two LVs (Figure 3a, Table 1). SSD has sufficient details to be unscrambled into 40 CovMs.

Figure 3a shows that SLAI-SSD>RWD predictions inside the RI (5.5–19.5 × $10^{9}$ cell/L) exhibit significant dispersion, as well as in the ASVCP High WBC clinical decision interval (SE = 5.837 × $10^{9}$ cell/L; Figure 3a). Higher errors were observed at higher WBC levels (>30 × $10^{9}$ cell/L), due to the lack of better representativity at these levels and higher errors in the quantification of automated hematology. Similarly to LocPLS, joining RWD and SSD, the SLAI-RWD and SSD model significantly reduces the error at high WBC values (Figure 3b), with a significant improvement in WBC prediction of RWD (R = 0.8397, MAPE = 32.25%) and SSD (R = 0.7723, MAPE = 25.86%), making it possible to increase the detail to 60 CovMs with one LV.

### 3.4. Bias–Variance Analysis

**Figure 4:**
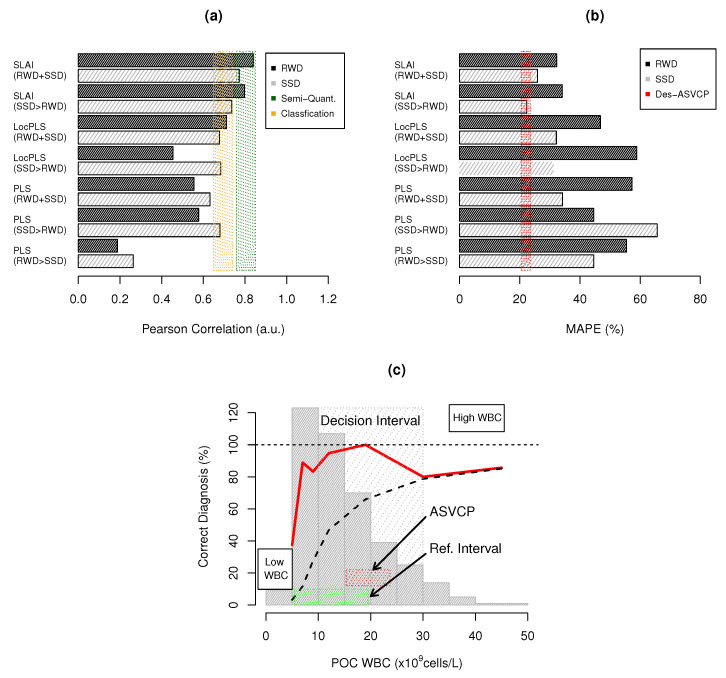
WBC quantification benchmarks: (**a**) Pearson correlation coefficient for RWD and SSD, where shaded rectangles represent semi-quantitative (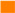) and quantitative (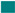) results; (**b**) MAPE benchmarks where (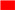) represents the ASVCP TAE for WBCs (21.45%); and (**c**) POC WBC diagnostic capacity: (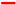) percentage of correct diagnoses within WBC value interval, (**– –**) accumulated percentage of correct diagnosis, (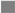) WBC histogram distibution; and RI for WBC (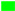) and (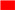) TAE diagnosis error tolerance.

Spectral POC linearity (Pearson R) and total error (MAPE) benchmarks are compared in Figure 4a,b. PLS is unable to provide qualitative or quantitative correlations, as R is below 0.65 for PLS-RWD, PLS-SSD>RWD and PLS-RWD>SSD. LocPLS has a significant increase in R, but WBC quantifications are qualitative only when RWD and SSD are used together. LocPLS also averages existing CovMs in a local group to build an ensemble of PLS models, but is still unable to unscramble the different types of interferences present in the knowledgebase (Figure 4a). SLAI has a semi-quantitative correlation (R ≃ 0.75–0.85). SLAI-SSD>RWD attains R = 0.7975, and, when using the full dataset, SLAI-RWD+SSD obtains R = 0.8397. SLAI unscrambles SSD and RWD into one LV CovMs.

Figure 4b shows the total error in WBCs for RWD and SSD under the different modeling approaches. All models were unable to obtain a TE below the TAE desired by ASVCP (TE ∼ 21.45%). PLS and LocPLS models have a high TE: (i) PLS (MAPE ∼ 44.56–67.79%) and (ii) LocPLS (MAPE ∼ 46.78–58.89%). SLAI attains a semi-quantitative TE of 32.15–34.13% in RWD, nearer the TAE.

Table 2 presents the percentage of cases with optimal, desired and acceptable TE, inside and outside the RI. PLS-SSD>RWD attains 39.06% and PLS-RWD+SSD attains 42.19% when predicting the RWD. PLS has a similar performance in predicting RWD (42.19%) and SSD (47.77%) when used together (PLS-RWD+SSD), where the results inside the RI are significantly better than those outside the RI. LocPLS-RWD+SSD has 46.88% RWD acceptable results inside the RI and 36.67% outside the RI. LocPLS-SSD>RWD had poor results (29.69%) but presented 52.68% inside and 52.63% outside the RI for SSD (Table 2).

SLAI has the highest values of acceptable TE for RWD inside the RI, 50.00% using SLAI-SSD>RWD and 54.69% for SLAI-RWD+SSD. It also provides the lowest TE outside the RI for RWD (43.33% and 50.00%). The TE outside and inside RI are very consistent in SL-AI when using only SSD or RWD with SSD (Table 2), decreasing from 54.69% to 50.00% in RWD and from 58.03% to 54.91% when not using the combination of RWD and SSD. Despite the SLAI performance, it cannot attain the TAE for WBCs of 21.45% [25]. This limit can be considered optimistic for today’s state-of-the-art hematology, as state-of-the-art WBC and differentiated counts still have significant errors, and a wider decision interval must be considered in clinical practice [53]. Figure 4c presents the percentage of correctly diagnosed WBC cases and the percentage of accumulated diagnosed WBC cases for SLAI-SSD>RWD for each WBC level. The percentage of correctly diagnosed cases inside the RI ranges from 83.33% to 100% inside the RI, and 90% within the diagnostic error margin of high WBC levels. The diagnostic accuracy reaches 80% for extreme cases above 30 × $10^{9}$ cell/L.

The study reveals high representativity within the RI (5.5–19.5 × $10^{9}$ cell/L), compared to extremely low or high levels above and below the RI. Besides the low representativity of abnormal values, extreme WBC values are associated with analytical errors in automated hemogram machines, due to the presence of abnormal cells or cell aggregates (e.g., platelet aggregates, parasitic inclusions, nuclear segmentation), which can be recognised as different types of WBCs, due to the similar scattering angles. In contrast, the high accuracy of the POC is found in the region of 7 to 30 × $10^{9}$ cell/L, which incorporates both RI and decision tolerance for high WBC diagnosis.

### 3.5. CovMs Interpretation

**Figure 5:**
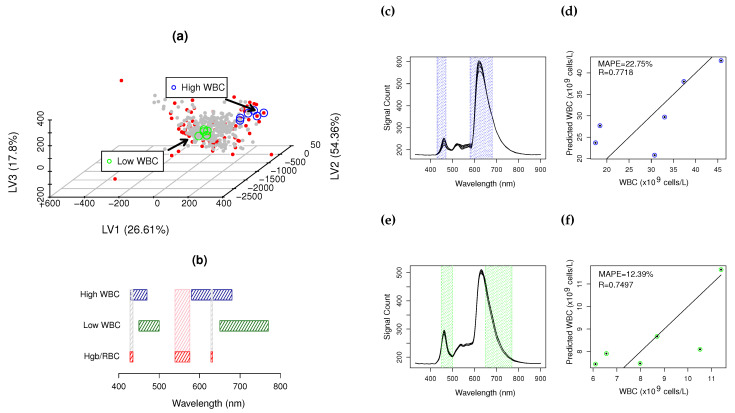
Representative low and high CovMs: (**a**) PLS scores space where (∘) and (∘) are low and high CovMs sample groups, • SSD blood samples, • RWD blood samples; (**b**) spectral ROIs used for the quantification of WBCs (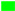 low and 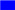 high CovM), (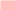) and (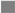) major and minor RBC/Hgb interference bands; (**c**,**e**) high and low CovM sample spectra and corresponding used ROIs to quantify WBCs; (**d**,**f**) prediction plot for high and low CovMs.

Figure 5 presents two CovMs at low and high WBC levels. Figure 5a shows the PLS scores space ( T ), where low and high WBCs are represented with green and blue circles, respectively. High WBC CovM spectra and quantification are presented in Figure 5c,d, with R = 0.7718 and MAPE = 22.75% within the range of 20–45.5 × $10^{9}$ cell/L. Low levels of WBC CovM spectra and quantification are presented in Figure 5e,f, with R = 0.7497 and MAPE = 0.7497, in the range of 6–12 × $10^{9}$ cell/L. High levels of WBCs are quantified using the regions of 430–470 nm and 580–680 nm, whereas low levels of WBC are quantified using 450–500 nm and 650–770 nm.

RBC/Hgb are dominant spectral features at low WBC values. WBCs absorb along all the UV-Vis spectra with more incidence in ROI 1 and 2, and RBC/Hgb information is mostly located in the ROI of 530–576 nm [1]. Low WBC CoMs use non-interferent RBC/Hgb spectral information, as presented in the interferogram of Figure 5b. A similar result is observed at high WBC levels, where minimal information overlapping occurs between WBCs and RBCs/Hgb (Figure 5b).

The apparent relationship between RBCs and WBCs, present in the global covariance (Figure 2), is not present at the CovM level, as it is an emergent property of the hemogram due to cell packing and scattering, only observable when the full knowledgebase is considered. At the CovM level, the interference mode is isolated, minimizing the major interferences, such as RBC/Hgb, by maximizing the WBC information into a single eigenvector.

#### Towards Information Specificity

The key principle of analytical methods is the use of specific information about a constituent, ensuring that unique information is used for its identification and quantification. Spectroscopy POC uses CovMs as a way to guarantee that constituents are quantified by specific interference or information. In a recent publication, we demonstrated that CovMs can exhibit orthogonal information to all other constituents, isolating specific information on N, P and K in nutrient solutions for hydroponics [40]. A similar result is demonstrated here for WBCs, where CovMs exhibit independent properties to RBCs/Hgb.

AI and chemometric methods risk the use of natural correlation in data by not isolating the unique information of a constituent. Information isolation using CovMs has the following advantages:

- Using independent spectral information allows us to diagnose natural correlations, which are highly common in complex biological information. A natural correlation between WBCs and RBCs can lead to dataset specificity but not WBC specificity; that is, WBCs are inferred by the intrinsic correlation of WBCs and RBCs. The isolation of WBC information provides a cause–effect model, preserving both information equivalence and statistical significance;
- The isolation of independent non-dominant information minimizes interference and maximizes WBC information. In this way, the sensitivity of WBC detection and quantification is increased (e.g., 85% at 7 × $10^{9}$ cell/L, Figure 4c).

Spectroscopy POC works in the spectral information domain. It is possible to assess unique and specific information about an analyte at a local CovM level, once information similarity is verified. Furthermore, if there is enough representativeness in the local information, it becomes feasible to isolate the unique information, which is a step towards ensuring analytical specificity in spectroscopy POC for complex biological samples.

## 4. Conclusions

Despite the lower quantities of WBCs in relation to RBCs (1:1000), their spectral information is significant due to scattering and absorbance, accounting for 0.5 to 22.5% of spectral information; this results in the natural relationship with RBCs in hemogram and spectroscopy score spaces due to scattering and absorbance. Data augmentation by hybridization is very effective for reproducing real-world data. It further increases the level of detail at each CovM level, allowing for the unscrambling of specific information related to WBCs, with minimal interference from RBCs or Hgb, whereby the isolated CovMs exhibit high orthogonality towards RBCs. Isolating CovMs that contain only WBC information significantly increases the quantification sensitivity because the spectroscopy signal maximizes the WBC variance. This allows for achieving correlations of 0.7975 to 0.8397, a mean absolute error of 32.25% to 34.13% and a diagnostic efficiency between 83–100% inside the reference interval (5.5 to 19.5 × $10^{9}$ cells/L) and 85.11% for cases with extremely high white blood cell counts. The obtained results are within the expected range of the existing state-of-the-art automated hematology analyzers in veterinary medicine.

## Figures and Tables

**Table 1:** Results benchmarks for PLS, LocPLS and SLAI for real-world data, and combination of real-world data and synthetic samples data for WBC quantification in cat blood.

		Real World Data (RWD)					Synthetic Sample Data (SSD)				
Method	nLV	R	R^2^	SE	MAPE (%)	*p*-Value	R	R^2^	SE	**MAPE** (%)	*p*-Value
PLS ^(1)^	2	0.1874	0.0350	5.498	55.45	0.001	–	–	–	–	–
PLS ^(2)^	15	0.5775	0.3336	6.109	44.56	1.12 × $10^{-9}$	0.6802	0.4626	5.135	65.65	<2 × $10^{-16}$
PLS ^(3)^	2	0.1874	0.0350	5.498	55.45	0.001	0.2634	0.0691	9.162	44.60	0.013
PLS ^(4)^	13	0.5553	0.3084	7.925	57.31	<2 × $10^{-16}$	0.6320	0.3995	9.736	34.18	<2 × $10^{-16}$
LocPLS ^(2)^	5	0.4546	0.2067	8.576	58.89	4.16 × $10^{-6}$	0.6840	0.4679	4.480	31.19	<2 × $10^{-16}$
LocPLS ^(4)^	7	0.7112	0.5059	6.673	46.78	<2 × $10^{-16}$	0.6782	0.4599	9.188	32.16	<2 × $10^{-16}$
SLAI ^(2)^	1–2	0.7975	0.6361	5.837	34.13	<2 × $10^{-16}$	0.7369	0.5460	8.492	22.35	<2 × $10^{-16}$
SLAI ^(4)^	1	0.8397	0.7051	5.192	32.25	<2 × $10^{-16}$	0.7723	0.5965	7.947	25.86	<2 × $10^{-16}$

^(1)^ Cross-validation optimization and hold-out metrics calculation using RWD. ^(2)^ Cross-validation optimization using SSD and hold-out metrics calculation using RWD. ^(3)^ Cross-validation optimization using RWD and hold-out metrics calculation using SSD. ^(4)^ Cross-validation optimization and hold-out metrics calculation using RWD and SSD.

**Table 2:** WBC bias analysis of WBCs in cat blood for spectroscopy POC—percentage of results in optimal, desired and acceptable categories.

	Real World Data						Synthetic Sample Data					
	% Inside RI			% Outside RI			% Inside RI			% Outside RI		
Method	Opt	Des	Accep	Opt	Des	Accep	Opt	Des	Accep	Opt	Des	Accep
PLS _RWD_^(1)^	7.81	14.06	26.56	0.00	0.00	10.00	na	na	na	na	na	na
PLS _SSD>RWD_^(2)^	21.88	29.69	**39.06**	10.00	16.67	20.00	15.63	29.02	**46.43**	13.16	22.37	**43.42**
PLS _RWD>SSD_^(3)^	7.81	14.06	26.56	0.00	0.00	10.00	16.07	25.89	34.38	1.32	2.63	9.21
PLS _SSD+RWD_^(4)^	10.94	26.56	**42.19**	3.33	10.00	13.33	13.84	29.91	**47.77**	9.21	22.37	26.32
LocPLS _SSD>RWD_^(2)^	14.06	25.00	29.69	13.33	16.67	20.00	23.21	**41.52**	**52.68**	21.05	35.53	**52.63**
LocPLS _RWD+SSD_^(4)^	15.63	32.81	**46.88**	16.67	23.33	36.67	19.20	32.14	**45.98**	14.47	32.89	**44.74**
SLAI _SSD>RWD_^(2)^	20.31	34.38	**50.00**	13.33	30.00	**43.33**	23.66	38.39	**54.91**	17.11	26.32	**43.42**
SLAI _RWD+SSD_^(4)^	31.25	**42.19**	**54.69**	13.33	26.67	**50.00**	24.12	**41.07**	**58.03**	30.26	35.52	**46.05**

^(1)^ Cross-validation optimization and hold-out metrics calculation using RWD. ^(2)^ Cross-validation optimization using SSD and hold-out metrics calculation using RWD. ^(3)^ Cross-validation optimization using RWD and hold-out metrics calculation using SSD. ^(4)^ Cross-validation optimization and hold-out metrics calculation using RWD and SSD. Highlighted bold values are considered are consedered best in class.

## Supplementary Materials

The following are available online at www.mdpi.com/xxx/s1: Figure S1: Spectral POC for Cat blood analysis: (1) widrawal of 10 μL of blood from ear lobe; (2) Injecting blood sample into capsule system; (3) plug-in the capsule into the POC probe; and (4) using IoT software to control POC measurement and record spectra for WBC calculation; Figure S2: Cat blood spectra: (a) real-world samples and (b) synthetic samples obtained by mixture of two samples spectra.

## Abbreviations

The following abbreviations are used in this manuscript:

ASVCP	American Society of Veterinary Clinical Pathology
BBL	Beer–Lambert Law
Bil	Bilirubin
BT	Blind Testing
CovM	Covariance Mode
CV	Cross-validation
EDTA	Ethylenediaminetetraacetic Acid
Hgb	Hemoglobin
HO	Hold out samples
HTC	Hematocrit
IoT	Internet of Things
LocPLS	Local Partial Least Squares
LV	Latent Variables
MAPE	Mean Absolute Percentage Error
PCV	Packed Cell Volume
PLS	Partial Least Squares
PLT	Platelets
POC	Point-of-care
R	Pearson Correlation
RBCs	Red Blood Cells
RI	Reference Interval
ROI	Region of Interest
RWD	Real World Data
SLAI	Self-learning Artificial Intelligence
SSD	Synthetic Spectroscopy Data
TAE	Total Allowable Error
TE	Total Error
TV	Target Values
UV-Vis	Ultraviolet–visible
Vis-SWNIR	Visible Short-Wave Infrared
WBCs	White Blood Cells
