# Supplementary material for: Reagentless Vis-NIR Spectroscopy Point-of-Care for Feline Total White Blood Cell Counts
Source: Biosensors (Basel). 2024 Jan 19;14(1):0. doi: 10.3390/bios14010053 (PMC11154536; doi:10.3390/bios14010053)
Supplement: Supplementary file 1 [file biosensors-14-00053-s001.zip › biosensors-2799131-supplementary.pdf]

Supplementary material

Complementary figures

The following figure presents complementary information cited in the manuscript.

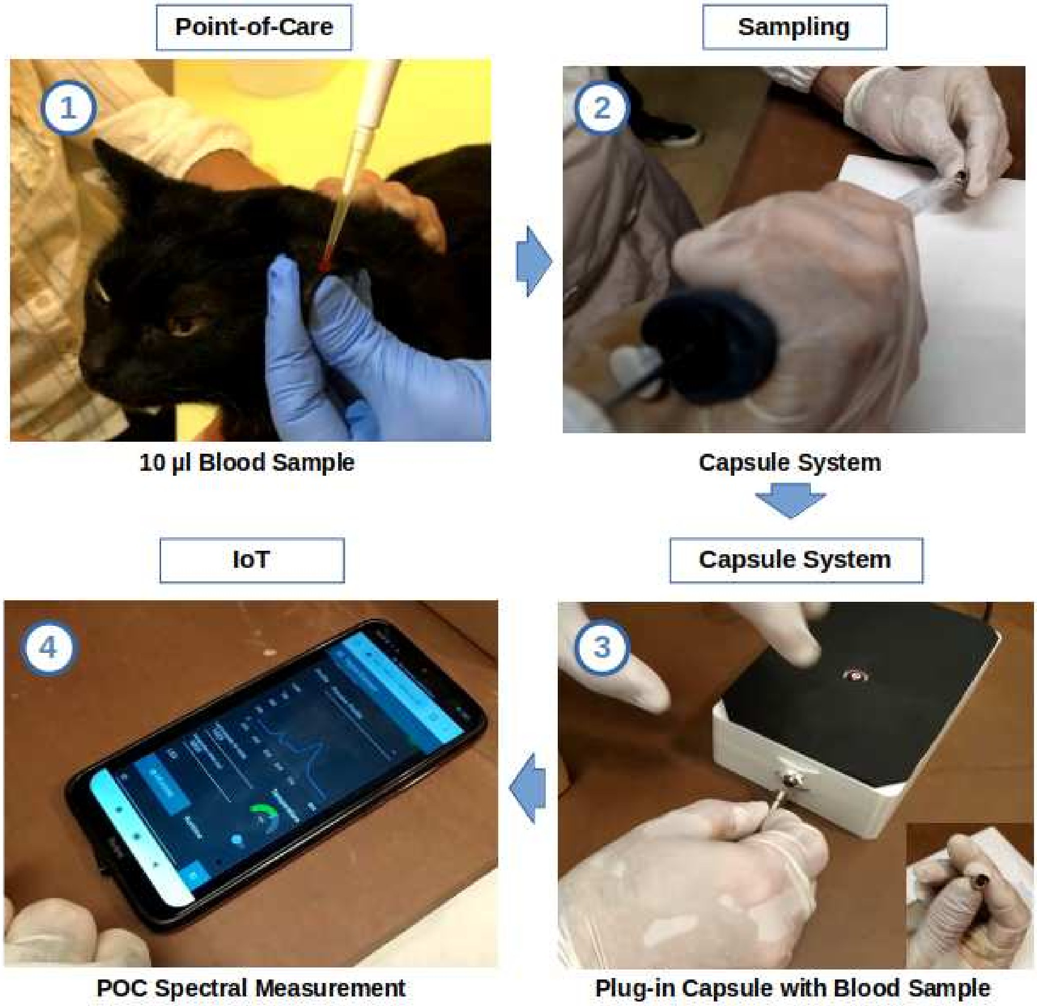

Figure S1. Spectral POC for Cat blood analysis: (1) widrawal of 10μL of blood from ear lobe; (2) Injecting blood sample into capsule system; (3) plug-in the capsule into the POC probe; and (4) using IoT software to control POC measurement and record spectra for WBC calculation.

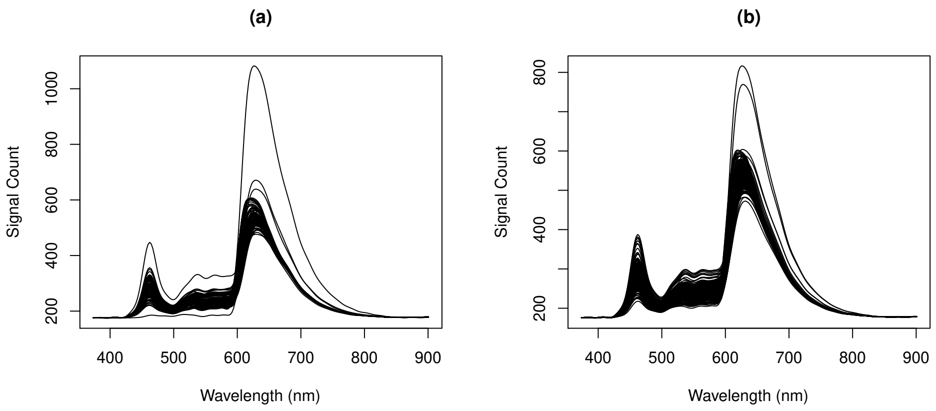

Figure S2. Cat blood spectra: (a) real-world samples and (b) sythetic samples obtained by mixture of two samples spectra.
